# Supplementary material for: Nociceptive interneurons control modular motor pathways to promote escape behavior in Drosophila
Source: eLife. 2018 Mar 12;7:e26016. doi: 10.7554/eLife.26016 (PMC5869015; doi:10.7554/eLife.26016)
Supplement: Supplementary file 1. [file elife-26016-supp1.pdf]

Main Figure genotypes:

Figure 1:

(B) *412-Gal4,UAS-CD4:tdTomato* (C-C') *hsFLP;Sp or CyO/+;412-Gal4/ UAS>CD2>CD8-GFP* (D) *+;tsh-LexA,8X-LexAopFLP; 10X-UAS-myr:GFP/412-Gal4* (E) *tsh-Gal80/+;412-Gal4, UAS-mCD8:GFP* (F) *tub>Gal80>;tshLexA,8X-LexAopFLP; 10X-UAS-myr:GFP/412-Gal4* (G) (i) *tsh-LexA,8X-LexAopFLP;412-Gal4/+* (ii) *UAS-dTrpA1/+;412-Gal4/+* (iii) *tsh-Gal80/UAS-dTrpA1;412-Gal4/+* (iv) *tub>Gal80>;tsh-LexA,8X-LexAopFLP; UAS-dTrpA1/412-Gal4* (J-M)) (i) *tsh-LexA,8X-LexAopFLP;412-Gal4/+* (ii) *ppk*$^{1.9}$*-Gal4/+;UAS-dTrpA1/+*(iii) *tub>Gal80>;tsh-LexA,8X-LexAopFLP; UAS-dTrpA1/412-Gal4*

Figure 2:

(A-E) *UAS-ReaChR/PPK1.9-Gal4* (C-D) *UAS-ReaChR/+:412-Gal4/+* (E) (i) *UAS-ReaChR/PPK1.9-Gal4* (ii) *UAS-ReaChR/+:412-Gal4/+*

Figure 3:

(A-C) *20X-UAS-IVS-GCaMP6m/+;412-Gal4* (D-F) (i) *20X-UAS-IVS-GCaMP6m/+;412-Gal4 /+* (ii) *20X-UAS-IVS-GCaMP6m/R38A10-LexA;412-Gal4 /13X-LexAop2-IVS-TNT::HA* (G) *w-; Sp or Cyo/R38A10-LexA; 13XLexAop2-IVS-myr:GFP/+* (H) (i) *38A10-LexA/+* (iii) *R38A10-LexA/+;13XLexAop2-IVS-TNT::HA /+* (I) (i) *UAS-dTrpA1/+;412-Gal4/+* (ii) *R38A10-LexA/UAS-dTrpA1;412-Gal4/13X-LexAop2-IVS-TNT::HA*

Figure 4:

Genotypes: (A, D-I) *R70F01-LexA/8X-Aop2FLPL; 412-Gal4/UAS>Stop>Kir*$^{2.1}$*-GFP* (C) (i) *8X-Aop2FLPL/+;10X-UAS>Stop>Kir2.1-GFP/+* (ii) *R70F01-LexA/8X-Aop2FLPL; 412-Gal4/UAS>Stop>Kir*$^{2.1}$*-GFP*

Figure 5:

Circuit diagram: (G) A1 Right/A1 Left: Class IV group: ddaC, v'ada, vdaB; Class III group ddaF, ddaA, ldaB, v'pda, vdaD; exernal sensory group: ventral'es, lesA

Figure 6:

Circuit diagram: (C) A1 right/A1 left: A10f, A09l; A4 right/A 4 left: A09e; A5 right/A5 left: A09e; Projection neuron: TePn05 (E) A1 right/A1 left: A09l, A27k, A01d-3, A18l, A02g, A02e, A03g, A27j, A07c4, A01c, LT1

Figure 7:

(B-E) *R69E06-LexAp65/+;20xUAS-CsChrimson-mCherry,13xLexAop2-IVS-GCaMP6s-p10/412-Gal4* (F-H) (i) *LexAop-Kir2.1/+;412-Gal4/UAS-dTrpA* (ii) *LexAop-Kir2.1/16e11-LexA;412-Gal4/UAS-dTrpA*

Circuit diagram: DnB group; A09l Down-and-Back (T3 A1 A2 A3 A4 A5 right/left); Basin- 2,4 group: A09a Basin-2 (A1 left, A2 right, A3 right, A4 right/left), A09c Basin-4 (A1 left/right, A3 left, A4 left); Basin-3 group: A09g Basin-3 (A1 right/left, A4 right); A09e group: A09e (A4 right/left, A5 right/left); TePn05 group: TePn05 class-IV related projection C (Right/Left); A02o Wave (T3 A1 A2 A3 A5 right/left, A4 A6 Left); A23g group: A23g (A1 right/left, A2 right/left); A05q group: A05q (A1 right/left); Goro group: Goro (T2 right/left)
